# NRX-101 (D-Cycloserine + Lurasidone) Is Active against Drug-Resistant Urinary Pathogens In Vitro

**DOI:** 10.3390/antibiotics13040308

**Published:** 2024-03-28

**Authors:** Michael T. Sapko, Michael Manyak, Riccardo Panicucci, Jonathan C. Javitt

**Affiliations:** 1 NRx Pharmaceuticals, 1201 N Market St, Suite 111, Wilmington, DE 19801, USA; 2 Department of Urology, George Washington University, 900 23rd Street NW, Washington, DC 20037, USA; 3 Department of Ophthalmology, Johns Hopkins University, 1800 Orleans St, Baltimore, MD 21287, USA

**Keywords:** D-cycloserine, complicated urinary tract infections, susceptibility testing, antimicrobials, NRX-101, multidrug resistance

## Abstract

D-Cycloserine (DCS) is a broad-spectrum antibiotic that is currently FDA-approved to treat tuberculosis (TB) disease and urinary tract infection (UTI). Despite numerous reports showing good clinical efficacy, DCS fell out of favor as a UTI treatment because of its propensity to cause side effects. NRX-101, a fixed-dose combination of DCS and lurasidone, has been awarded Qualified Infectious Disease Product and Fast Track Designation by the FDA. In this study, we tested NRX-101 against the urinary tract pathogens *Escherichia coli*, *Pseudomonas aeruginosa*, *Klebsiella pneumoniae*, and *Acinetobacter baumannii* in cation-adjusted Mueller–Hinton broth (caMHB) and artificial urine media (AUM). Several strains were multidrug resistant. Test compounds were serially diluted in broth/media. Minimum inhibitory concentration (MIC) was defined as the lowest concentration of the test compound at which no bacterial growth was observed. DCS exhibited antibacterial efficacy against all strains tested while lurasidone did not appreciably affect the antibacterial action of DCS in vitro. In AUM, the MICs ranged from 128 to 512 mcg/mL for both DCS and NRX-101. In caMHB, MICs ranged from 8 to 1024 mcg/mL for NRX-101 and 32 to 512 mcg/mL for DCS alone. Our data confirm that DCS has antibacterial activity against reference and drug-resistant urinary pathogens. Furthermore, lurasidone does not interfere with DCS’s antimicrobial action in vitro. These results support the clinical development of NRX-101 as a treatment for complicated urinary tract infections.

## 1. Introduction

D-Cycloserine (DCS) is a broad-spectrum antibiotic that is currently FDA-approved to treat pulmonary or extrapulmonary tuberculosis (TB) disease (active TB) and urinary tract infection (UTI) [1]. In the 1950s and 1960s, DCS was used to treat various types of infections including UTIs, with numerous reports showing its clinical efficacy [2,3,4,5]. Landes et al., for example, reported good clinical efficacy against a wide variety of urinary tract pathogens including *Escherichia coli, Pseudomonas* spp., *Aerobacter aerogenes* (renamed *Enterobacter aerogenes*), various *Proteus* strains, *Staphylococcus* spp. and *Streptococcus* spp. The use of DCS as an antibiotic for the treatment of UTI fell out of favor in the 1970s due to its potential to cause central nervous system (CNS) side effects. However, because the incidence of multidrug-resistant urinary tract pathogens [6] is outpacing the development of new antimicrobial agents [7], several authors have begun to re-examine the use of older antibiotics such as DCS [8,9]. In January 2024, a fixed-dose combination of DCS and lurasidone was awarded Qualified Infectious Disease Product Designation by the US FDA for the treatment of complicated urinary tract infection (cUTI) and pyelonephritis.

D-cycloserine (DCS) inhibits two sequential enzymes in the bacterial cell wall peptidoglycan biosynthetic pathway, which forms the dipeptide D-alanyl-D-alanine (D-Ala-D-Ala) [10]. DCS targets alanine racemase to form an aromatized DCS-pyridoxal-phosphate (PLP) adduct, which irreversibly blocks alanine racemase activity [11]. DCS also competitively and reversibly inhibits D-Ala-D-Ala ligase [10]. DCS also antagonizes a third bacterial enzyme, D-amino acid dehydrogenase [8].

DCS is a partial agonist of the human NMDA receptor and crosses the blood–brain barrier [12]. This binding and activation profile has attracted vigorous research interest in DCS as a treatment for acute suicidality in bipolar depression, post-traumatic stress disorder, and chronic pain. However, DCS action at NMDA receptors is also likely the cause of rare but potentially treatment-limiting side effects such as hallucinations [13]. Lurasidone is a full antagonist at dopamine D2 and serotonin 5-HT2A and 5-HT7 receptors [14]. Nonclinical and limited clinical testing conducted to date suggests that DCS and lurasidone ameliorate the known side effects of the other. As a combined D_2_/5HT_2A_ antagonist, lurasidone may have the ability to reverse the psychotomimetic effects of DCS, and DCS, in turn, appears to block the anxiogenic effects of lurasidone [15,16]. Therefore, we are investigating the possibility that lurasidone may ameliorate the CNS effects of DCS when it is used as an antimicrobial agent against urinary tract pathogens.

NRX-101 is a fixed-dose combination of DCS and lurasidone that is being developed for various CNS indications. In the current study, we show that DCS alone and NRX-101 (DCS plus lurasidone) are active against common urinary tract bacterial pathogens, including microbes that are highly resistant to standard antimicrobials. We also show that lurasidone does not interfere with the antimicrobial effect of DCS.

## 2. Results

All bacterial isolates grew in CaMHB. All bacterial isolates except *Acinetobacter baumannii* 19606 grew in AUM. MIC results in CaMHB and AUM are shown in Table 1 and Table 2, respectively. DCS showed activity against all the bacterial isolates tested. Lurasidone alone had no antibacterial activity at the concentrations tested in either medium. When tested as a combination, lurasidone did not appreciably affect the antibacterial efficacy of DCS. MICs of DCS were often higher for bacterial isolates grown in AUM compared to those grown in caMHB. This effect was most apparent for the *E. coli* and *Pseudomonas aeruginosa* strains tested. Reference antibiotics performed as expected.

## 3. Discussion

Based on the above results, the US Food and Drug Administration has awarded Qualified Infectious Disease Product and Fast Track Designation to NRX-101, a fixed-dose combination of DCS and lurasidone for the treatment of cUTI, including acute pyelonephritis. 

DCS and DCS + lurasidone (NRX-101) showed antibacterial activity against various strains of *E. coli*, *P. aeruginosa*, *K. pneumoniae*, and *A. baumannii* in both caMHB and AUM. In AUM, the MICs ranged from 128 to 512 mcg/mL for both DCS and NRX-101. In caMHB, MICs ranged from 8 to 1024 mcg/mL for NRX-101 and 32 to 512 for DCS alone. Kugathasan et al. tested DCS against 500 urinary pathogens and reported a range of MICs from 8 to 128 mcg/mL in Mueller–Hinton broth [9]. That group reported the same range for the subset of 411 *E. coli* isolates within the larger test group. Our range of MICs against *E. coli* isolates is 32 to 128 mcg/mL. While our group and Kugathasan’s groups both show the antibacterial efficacy of DCS, we observed lower potency of the antibiotic in vitro. One possible explanation is that in the large assortment of urinary pathogens Kugathasan tested, certain strains not included in our study were particularly susceptible to DCS. 

Our results are congruent with those of Kugathasan et al. [9], who demonstrated a similar range of MICs against 182 trimethoprim and 24 third-generation cephalosporin-resistant isolates. This is to be expected since DCS exerts its antibacterial effect by blocking various enzymes in the bacterial cell wall peptidoglycan biosynthetic pathway (alanine racemase activity, D-Ala-D-Ala ligase, and D-amino acid dehydrogenase). This mechanism is distinct from that of trimethoprim, which blocks the reduction of dihydrofolate to tetrahydrofolate. Similarly, the DCS mechanism is distinct from the beta-lactam action of third-generation cephalosporins against peptidoglycan transpeptidase. This distinct mechanism of action is relevant in light of the high rate of antimicrobial resistance among urinary tract pathogen isolates.

Because DCS competes with d-alanine in the peptidoglycan biosynthetic pathway to exert its antibacterial effect, various groups have suggested that DCS be tested in alanine-free media [9,17,18] because the presence of alanine leads to “falsely elevated MICs” [9]. Kugathasan et al. reported a MIC as low as 0.12 mcg/mL for DCS against urinary pathogenic isolates in an alanine-free, Minimal Salts medium [9]. Indeed, the presence of alanine in culture media may explain why authors in the 1950s and 1960s reported greater clinical efficacy with DCS than one would expect from corresponding MICs determined from the patient’s bacterial isolates [3]. However, we suggest that alanine-free media provides an estimate of the MIC of DCS under ideal conditions, but may not reflect the MIC under real-world conditions. Both Mueller–Hinton broth, derived from beef extract, and the artificial urine medium used in our study contain alanine [19], as does human urine [20].

DCS is excreted mostly unchanged in the urine [1]. Welch et al. reported that a single oral dose of 1000 mg of DCS caused DCS levels in the urine to peak at approximately 200 mcg/mL within 8 h [21]. From the same study, a daily dose of 2000 mg (500 mg doses of DCS given every 6 h) achieved a peak urine concentration of 800 mcg/mL at 72 h [21]. When combined with lurasidone, daily DCS doses as high as 950 mg have been well tolerated in our clinical trial [22]. Urine levels of DCS are likely to exceed even the highest MICs for urinary pathogens reported here (512 mcg/mL) and elsewhere [9]. We are currently performing a pharmacokinetic study of NRX-101 in plasma and urine to more precisely characterize urine levels of DCS after oral administration using modern analytical techniques.

One limitation of our study is that we used a basic serial dilution MIC protocol. To identify the relative contributions of each component of NRX-101, we would need to perform a checkerboard assay. Our primary concern in these initial experiments was to determine if lurasidone interfered with the antibacterial action of DCS. Indeed, lurasidone did not influence the antibacterial efficacy at any dose tested. It should also be noted that the concentration of lurasidone tested in in vitro studies, 142.3 mcg/mL, is far higher than would be expected to be found in the urine of patients taking therapeutic doses of lurasidone, which is 5.7 to 260.1 ng/mL, three orders of magnitude lower [23]. Thus, the inclusion of lurasidone in NRX-101 to mitigate CNS side effects from DCS is not expected to interfere with the anti-infective action of DCS in the urinary tract. The addition of lurasidone, however, is likely to offset the mild CNS effects of DCS, and those effects have not been observed in studies where high-dose DCS has been used to treat bipolar depression [22].

## 4. Materials and Methods

### 4.1. Bacterial Isolates

A total of 13 bacterial isolates were tested: 6 *Escherichia coli*, 4 *Pseudomonas aeruginosa*, 1 *Klebsiella pneumoniae*, and 2 *Acinetobacter baumannii* strains. Specific strains and their antibiotic susceptibilities are listed in Appendix A (Table A1).

### 4.2. Antimicrobial Susceptibility Testing

Antimicrobial susceptibility testing was carried out by Charles River Laboratories (Portishead) in accordance with CLSI guidelines M07-A10. Sources of materials are listed in Appendix A (Table A2). Bacterial inoculum was adjusted to the equivalent OD_600_ of 0.5 McFarland standard and added to plates at one concentration (1:1, *v*:*v*). Assays were carried out in both cation-adjusted Mueller–Hinton broth (caMHB; Millipore Sigma, Darmstadt, Germany) and artificial urine media (AUM; Biochemazone, AB, Canada). Bacteria grown in caMHB and in AUM required 24 and 48 h incubation time, respectively. Further, 96-well plates with triplicate technical replicates of each condition were incubated at 37 °C under aerobic conditions. To assess the minimum inhibitory concentrations (MICs), test compounds were serially diluted in broth/media to 12 dilutions. Each strain was tested for its susceptibility to d-cycloserine (DCS; Sigma-Aldrich, Darmstadt, Germany), lurasidone (MedChemExpress, Monmouth Junction, NJ, USA), DCS + lurasidone, and one antibiotic against which the test strain is known to be susceptible. Control antibiotics gentamicin sulfate salt, colistin sulfate salt, ciprofloxacin, and tobramycin were obtained from Sigma-Aldrich. Each strain was also allowed to grow without the presence of any test compound or antibiotic. Bacterial growth was determined visually. MIC was defined as the lowest concentration (i.e., greatest dilution) of the test compound at which no bacterial growth was observed. Sterility checks were performed by adding media with inoculum to each well per guidelines. Back-plating was performed to confirm pure inoculum.

## 5. Conclusions

NRX-101, a fixed-dose combination of DCS and lurasidone, is active against the urinary tract pathogens *E. coli*, *P. aeruginosa*, *K. pneumoniae*, and *A. baumannii* grown in vitro, including several multidrug-resistant strains. DCS exhibited antibacterial efficacy against all strains tested while lurasidone did not appreciably affect the antibacterial action of DCS in vitro. Urine levels of DCS are likely to exceed the MICs for urinary pathogens. These results support the clinical development of NRX-101 as a treatment for complicated urinary tract infections.

## Figures and Tables

**Table 1:** Minimum inhibitory concentrations of DCS and lurasidone in cation-adjusted Mueller–Hinton broth.

Strain	Reference Antibiotic (μg/mL)	Lurasidone (μg/mL)	DCS (μg/mL)	DCS + Lurasidone (μg/mL)
*E. coli 35218*	2 ^a^	>142.3	32	32
*E. coli 25922*	1 ^a^	>142.3	32	32
*E. coli 700928*	2–4 ^a^	>142.3	32	8
*E. coli 700336*	2 ^a^	>142.3	32	32
*E. coli 2469*	0.03–0.062 ^b^	>142.3	64–128	32
*E. coli* Xen 16	1 ^a^	>142.3	64	32
*P. aeruginosa* PA01	1 ^c^	>142.3	256	128
*P. aeruginosa 27853*	0.5 ^c^	>142.3	256	128
*P. aeruginosa* Xen 41	0.5 ^c^	>142.3	128	64
*P. aeruginosa* BAA 3105	64 ^c^	>142.3	128	128
*K. pneumoniae* 1705	1 ^a^	>142.3	128	128
*A. baumannii 19606*	32 ^a^	No growth	512	256
*A. baumannii 1605*	64 ^d^	>142.3	512	1024

^a^ Gentamicin; ^b^ Colistin; ^c^ Tobramycin; ^d^ Ciprofloxacin.

**Table 2:** Minimum inhibitory concentrations of DCS and lurasidone in artificial urine media.

Strain	Reference Antibiotic (μg/mL)	Lurasidone (μg/mL)	DCS (μg/mL)	DCS + Lurasidone (μg/mL)
*E. coli 35218*	32 ^a^	>142.3	256	128
*E. coli 25922*	256 ^a^	>142.3	256	256
*E. coli 700928*	32 ^a^	>142.3	256	256
*E. coli 700336*	128 ^a^	>142.3	128	256
*E. coli 2469*	1 ^b^	>142.3	>256	>256
*E. coli* Xen 16	>256 ^a^	>142.3	256	256
*P. aeruginosa* PA01	32 ^c^	>142.3	512	512
*P. aeruginosa 27853*	32 ^c^	>142.3	512	512
*P. aeruginosa* Xen 41	32 ^c^	>142.3	512	128
*P. aeruginosa* BAA 3105	16 ^c^	>142.3	128	128–256
*K. pneumoniae* 1705	>256 ^a^	>142.3	128	256
*A. baumannii 19606*	No growth	No growth	No growth	No growth
*A. baumannii 1605*	32 ^d^	>142.3	512	256

^a^ Gentamicin; ^b^ Colistin; ^c^ Tobramycin; ^d^ Ciprofloxacin.

## Data Availability

The original contributions presented in the study are included in the article; further inquiries can be directed to the corresponding authors.

## Appendix A

**Table A1:** Strains tested and their antimicrobial resistance profiles.

Strain	Antimicrobial Resistance
*Escherichia coli* ATCC 35218	Reference strain
*Escherichia coli* ATCC 25922	Reference strain
*Escherichia coli* ATCC 700928	Uropathogenic strain
*Escherichia coli* ATCC 700336	Uropathogenic strain
*Escherichia coli* ATCC BAA-2469	Carbepenem-resistant (imipenem and ertapenem)
*Escherichia coli* Xen16	Resistant to ampicillin; parental strain WS2583
*Pseudomonas aeruginosa* PAO1 (ATCC 15692)	Reference strain
*Pseudomonas aeruginosa* ATCC 27853	Reference strain
*Pseudomonas aeruginosa* Xen 41	Parental strain PAO1
*Pseudomonas aeruginosa* ATCC BAA-3105	Global Priority Superbug: resistant to cefepime, ceftazidime, ceftazidime-avibactam, ciprofloxacin, doripenem, imipenem, levofloxacin, meropenem
*Klebsiella pneumoniae* BAA-1705	Carbepenem-resistant (imipenem and ertapenem)
*Acinetobacter baumannii* ATCC 19606	Resistant to ampicillin, amoxicillin/clavulanic acid, cefazolin, cefoxitin, nitrofurantoin, trimethoprim, and trimethoprim/sulfamethoxazole
*Acinetobacter baumannii* ATCC BAA-1605	Resistant to ceftazidime, gentamicin, ticarcillin, piperacillin, aztreonam, cefepime, ciprofloxacin, imipenem, and meropenem.

**Table A2:** Key compounds and their sources.

Component.	Supplier	Cat #	Lot #	Stock Conc.
Cation-adjusted Muller–Hinton broth	Millipore Sigma	90922	102490943	
Artificial urine media	Biochemazone	B7103	B7103-0723C	
PBS (+/+)	Thermo Fisher Scientific	2669384	14040-083	
D-cycloserine	Sigma-Aldrich	30020	0000131087	20 mg/mL
Lurasidone	Medchem Express	HY-BOO32	CS-0866	246 mg/mL
Gentamicin sulfate salt	Sigma-Aldrich	G1264	0000212509	10 mg/mL
Tobramycin	Sigma-Aldrich	T4014	0000161605	10 mg/mL
Colistin sulfate salt	Sigma-Aldrich	C4461	0000129926	
Ciprofloxacin	Sigma-Aldrich	17850	116M4062V	10 mg/mL
